# Myoelectrical Manifestation of Fatigue Less Prominent in Patients with Cancer Related Fatigue

**DOI:** 10.1371/journal.pone.0083636

**Published:** 2013-12-31

**Authors:** Katarzyna Kisiel-Sajewicz, Vlodek Siemionow, Dilara Seyidova-Khoshknabi, Mellar P. Davis, Alexandria Wyant, Vinoth K. Ranganathan, Declan Walsh, Jin H. Yan, Juliet Hou, Guang H. Yue

**Affiliations:** 1 Department of Biomedical Engineering, the Lerner Research Institute, the Cleveland Clinic, Cleveland, Ohio, United States of America; 2 The Harry R. Horvitz Center for Palliative Medicine, the Taussig Cancer Center, the Cleveland Clinic, Cleveland, Ohio, United States of America; 3 Department of Physical Medicine and Rehabilitation, the Neurological Institute, the Cleveland Clinic, Cleveland, Ohio, United States of America; 4 Department of Kinesiology, Faculty of Physiotherapy, University School of Physical Education in Wroclaw, Wroclaw, Poland; 5 Institute of Affective and Social Neuroscience, Shenzhen University, Shenzhen; Department of Psychology, Tsinghuan University, Beijing, China; 6 Kessler Foundation Research Center, West Orange, New Jersey, United States of America; Cleveland Clinic Lerner Research Institute, United States of America

## Abstract

**Purpose:**

A lack of fatigue-related muscle contractile property changes at time of perceived physical exhaustion and greater central than peripheral fatigue detected by twitch interpolation technique have recently been reported in cancer survivors with fatigue symptoms. Based on these observations, it was hypothesized that compared to healthy people, myoelectrical manifestation of fatigue in the performing muscles would be less significant in these individuals while sustaining a prolonged motor task to self-perceived exhaustion (SPE) since their central fatigue was more prominent. The purpose of this study was to test this hypothesis by examining electromyographic (EMG) signal changes during fatiguing muscle performance.

**Methods:**

Twelve individuals who had advanced solid cancer and cancer-related fatigue (CRF), and 12 age- and gender-matched healthy controls performed a sustained elbow flexion at 30% maximal voluntary contraction till SPE. Amplitude and mean power frequency (MPF) of EMG signals of the biceps brachii, brachioradialis, and triceps brachii muscles were evaluated when the individuals experienced minimal, moderate, and severe fatigue.

**Results:**

CRF patients perceived physical “exhaustion” significantly sooner than the controls. The myoelectrical manifestation of muscular fatigue assessed by EMG amplitude and MPF was less significant in CRF than controls. The lower MPF even at minimal fatigue stage in CRF may indicate pathophysiologic condition of the muscle.

**Conclusions:**

CRF patients experience less myoelectrical manifestation of muscle fatigue than healthy individuals near the time of SPE. The data suggest that central nervous system fatigue plays a more important role in limiting endurance-type of motor performance in patients with CRF.

## Introduction

Fatigue and physical impairments are prevalent in cancer survivors [1]. Cancer-related fatigue (CRF) is a major factor that limits physical abilities in these individuals. CRF is one of the most prevalent symptoms in cancer survivors, which is thought to have the greatest adverse influence on quality of life, both during and following treatment [2], [3]. A better understanding of the mechanism of CRF is important in order to improve diagnosis, develop more targeted therapies, and promote physical well being in cancer patients. Although the etiology of CRF is poorly understood, a number of mechanisms are proposed, ranging from the central nervous system dysfunction to abnormal muscle metabolism [4], [5].

The degree of peripheral (muscle) fatigue is often determined by electrical stimulation in which one or more supermaximal-intensity electrical pulses are applied to a muscle or the nerve going into the muscle and measuring the evoked twich force response. During voluntary exercise the failure to maintain the required force depends on peripheral fatigue occurring distal to the point of stimulation and on central fatigue resulting from a failure to activate the muscle voluntarily [6]. A direct measure of peripheral fatigue is the change in the evoked twitch force immediately following the fatigue exercise relative to the same twitch force evoked before the fatigue exercise. If the twitch force is significantly smaller after than before the fatigue exercise, it reveals a significant loss of force generating capability of the muscle and indicates serious muscle fatigue. In addition, other parameters derived from the twitch force can show alterations in contractile properties of the muscle such as fatigue-induced slowing the rate of muscle contraction and relaxation [7]. A lack of fatigue-related muscle contractile property changes at the time of perceived physical exhaustion has recently been reported in cancer survivors with fatigue symptoms [8]. A recent study examined as well electrical stimulation-evoked muscle force during voluntarily generated sustained force from the biceps brachii [9]. CRF patients exhibited greater central than peripheral fatigue when performing a typical submaximal-level muscle contraction till self-perceived exhaustion (SPE) and task failure [9]. CRF patients felt exhausted and could no longer continue muscle activity, however, the muscle was still able to produce force by an external input (electrical stimulation), indicating that muscle fatigue was less at the time of SPE [9]. However, evidence of less prominent myoelectrical manifestation of muscle fatigue at the time of SPE in CRF measured by standard myoelectrical fatigue parameters has not been demonstrated.

Numerous studies designed to investigate myoelectric manifestation of muscular fatigue have used standard electromyographic (EMG) analysis to determine the degree of muscle fatigue in healthy individuals [10]–[14]. In the previous studies the analysis in time and frequency domains of surface EMG signals detected during fatiguing muscle contractions allowed assessment of myoelectric manifestation of muscle fatigue and were proved to be able to differentiate between subject groups [15] and muscles [10], [16]. Because of the difficulties in isolating so many factors (the motor cortex, the excitory drive, the control strategies of the spinal-upper and the α-lower motoneurons, the moteuron conduction properties, the neuromuscular transmission, the sarcolemmal excitability and conduction properties, the excitation-contraction coupling, the metabolic energy supply, and the contraction mechanisms) influencing the surface EMG signal during fatigue, particular experimental protocols have been developed to limit the neuromuscular system being influenced by a small number of factors when applying a given protocol [14]. When a prolonged low-level isometric voluntary contraction is sustained, the level of muscle fatigue relates to EMG amplitude [17], [18] and inversely relates to the median/mean frequency signals [19], [20]. When healthy individuals sustain a submaximal contraction to task failure, the amplitude of surface EMG increases due to the recruitment of additional motor units [21], [22], reduction in muscle fiber conduction velocity [13], and changes in the shape of intracellular action potentials [23]. Thus, if a muscle is not significantly fatigued during a motor task (such as the one performed by CRF patients) [24], EMG amplitude and frequency signals recorded from the patients would demonstrate insignificant myoelectrical manifestation of muscular fatigue and significant muscle reserve. Accordingly, the purpose of this study was to examine the level of myoelectric manifestations of muscle fatigue in CRF during a sustained submaximal contraction to task failure and compare this to normal individuals. It was hypothesized that the myoelectrical manifestation of muscular fatigue would be less prominent at the time of task failure in CRF compared to patients' perception and controls. Our data strongly support this hypothesis; the findings have been reported in abstract form [25].

## Methods

### Ethics Statement

This study was approved by Institutional Review Board at the Cleveland Clinic. All subjects has given written informed consent prior to participation.

### Subjects

Twelve patients (59.2±10.4 years old, body mass: 74.7±13.1 kg, height: 169±10 cm, BMI: 26.4±5.8, 8 women) with history of advanced solid cancer and CRF (Table 1) and 12 healthy controls (46.6±12.8 years old, body mass: 70.3±12.7 kg, height: 165±10 cm, BMI: 25.7±4.2, 9 women, all right handed) without a cancer history and with no known neurological, muscular and skeletal disorders or other conditions that would influence their sensorimotor performance participated in the study. Handedness of the subjects was assessed by the Edinburg Inventory [26]. Age was not significantly different between the two groups (*P*>0.05). CRF was assessed by the Brief Fatigue Inventory (BFI) [27]. CRF group reported significantly higher BFI fatigue scores (5.5±2.5) than the control group (0.9±1.1) for all BFI questions (*P*<0.05). No patient had had surgery or received chemo/radiation therapy within four weeks prior to the study. Eligible patients had a hemoglobin concentration >10 g/dl, and no clinical evidence of polyneuropathy, amyotrophy, or a myasthenic syndrome, by history review and medical examination. Significant pulmonary compromise as determined by oxygen dependence was an exclusion criterion for both groups. Patients and controls who were depressed or currently on psychostimulants or antidepressants were excluded. Subjects were evaluated by the screening physicians to exclude those (patients and controls) with depression. Patients with weight loss greater than 10% of pre-illness body weight were excluded.

**Table 1:** Demographic and clinical data of the cancer-related fatigue group.

Subject	Age (years)	Gender^1^	Dominant Hand^2^	BMI^3^ (kg/m^2^)	Cancer Stage	4ECOG
1	65	M	R	26.6	Angiosarcoma-IV	3
2	48	F	R	19.8	Thyroid-III	1
3	71	M	R	22.5	Colon-IV	2
4	48	M	L	21.7	Liver-IV	0
5	63	F	R	21.4	Peritoneum-IV	1
6	60	F	R	31.0	Chondrosarcoma-IV	1
7	81	M	R	23.2	Lung-IV	2
8	48	F	R	30.9	Ovarian-IV	2
9	64	F	R	19.6	Lung-III	1
10	48	F	R	37.2	Cervical-IV	2
11	57	F	R	31.6	Lymphoma-III	1
12	58	F	L	30.8	Lung-III	1

^1^ F - female, M- male

^2^ R - right, L - left

^3^ BMI  =  Body Mass Index

^4^ ECOG  =  Eastern Cooperative Oncology Group Score is used to assess how a patient's disease is progressing and how the disease affects the daily living abilities of the patient (0–5). A score of 0 is considered fully active, able to carry on all pre-disease performance without restriction and 5 is considered dead.

Details of patient demographics are provided in Tables 1. Details of controls demographics and experimental design are previously published in Yavuzsen et al. [9]. EMG data of 24 of 32 individuals in Yavuzsen et al. [9] were analyzed and those of the rest 12 were discarded due to excessive artifacts (caused by electrical stimulation during sustained contraction) in EMG signals (8 individuals). In addition, twitch force elicited by electrical stimulation of the biceps brachii muscle (before and immediately after the sustained contraction) in the 24 subjects was evaluated to learn whether muscle fatigue revealed by EMG changes is also recognized by twitch force amendments.

### Fatigue Motor Task

The patient and control groups followed exactly the same experimental protocols. All subjects performed a sustained isometric contraction of the dominant-arm flexor muscles at 30% of maximal voluntary contraction (MVC) till they felt exhausted (self-perceived exhaustion) and were no longer able to continue the contraction. Participants were vigorously verbally encouraged to continue the motor task for as long as possible. The motor task was terminated if the exerted force dropped 10% from the target for more than 3 s. Participants performed the motor task in a sitting position with the elbow joint flexed at ∼100° (180° = elbow joint fully extended) and the subject's forearm was fixed onto a supporting arm attached to the chair. The forearm was in the middle (neutral) position between pronation and supination of the hand. A horizontal cursor representing the target force was displayed on an oscilloscope. Participants were encouraged to maintain the exerted force (represented by the trace of an oscilloscope channel with the force input) to match the target for as long as possible.

### Elbow Flexion Force Measurement

Maximal voluntary contraction (MVC) force of the dominant-arm elbow flexion was measured using a force transducer (JR3 Universal Force-Moment sensor system, Woodland, CA) and then the 30% (target) force level was calculated based on the MVC force. The MVC force was measured both before and immediately after the sustained fatigue contraction (mean interruption between the time of task termination and initiation of the post-fatigue MVC was 18.5 ± 4 s). Elbow flexion force during the submaximal fatigue contraction was measured continuously by the same force transducer for the MVC force. Force signal was acquired by a data-acquisition system (1401 Plus, Cambridge Electronic Design, Ltd., Cambridge, UK), digitized at 100 samples/s, and stored on hard disk of a personal computer.

### EMG Measurement

Electromyographic (EMG) signals were recorded from the biceps brachii (BB) and brachioradialis (BR) (two elbow flexors), and triceps brachii (TB, the antagonist) muscles simultaneously using bipolar surface electrodes (Ag-AgCl, In Vivo Metric, Healdsburg, CA). Two electrodes were attached to the skin over the belly of each muscle in the direction of muscle fiber orientation. The distance between centers of the two electrodes was ∼3 cm with an 8-mm recording area for each electrode. A reference electrode was placed on the lateral epicondyle near the elbow joint. The skin was cleaned with alcohol wipes and the electrode cavity was filled with conducting gel (Signa Gel, Parker Laboratories, Inc., Fairfield, NJ) before attachment. EMG signals were amplified (X1000), band-pass filtered (3–500 Hz), digitized at 2000 samples/s, acquired by the 1401-Plus system, and saved in hard disk of the PC.

### Twitch Force Measurement

Twitch force (TF) was assessed before and immediately after the sustained contraction (fatigue motor task) to evaluated muscle force generating capability. Stimulation electrodes were attached to the skin overlying the BB muscle. Supramaximal-intensity single electrical pulses (1-ms duration) were applied through a digital stimulator (Grass S8800) to evoke TF. The voltage employed was at least 20% greater than that required to produce a maximum response. TF was measured using the same force transducer for the MVC force. Peak TF force (N) was quantified from baseline to peak of the TF.

### Data Processing and Analysis

#### Endurance time

The time duration from the moment when force reached the target to the point at which the sustained contraction was terminated was defined as endurance time. When subjects felt exhausted (SPE), they terminated the contraction leading to task failure.

#### Force analysis

**Figure 1:**
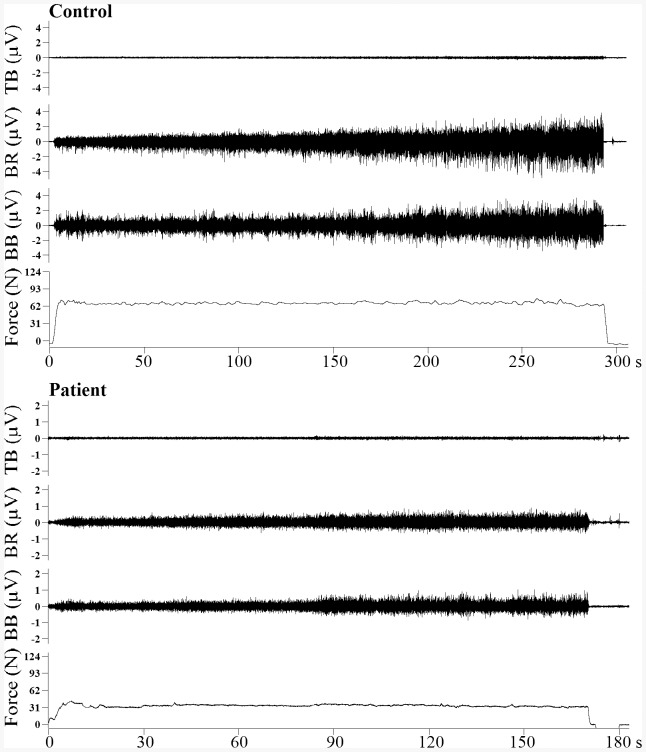
An illustration of raw EMG and force recordings from a control and CRF patient. The EMG increased gradually in all 3 muscles (biceps brachii - BB, brachioradialis - BR, and triceps brachii - TB) during and the force was maintained on target throughout the sustained contraction for both the control and CRF subjects.

Elbow flexion MVC force was measured from baseline to peak force and represented subjects' elbow flexion strength. Force recording during 30% MVC sustained contraction was divided into three (initial - minimal fatigue, middle - moderate fatigue, final - severe fatigue) periods. A 10-s epoch of force data was segmented from each of the three periods and average force was determined for each period. The first epoch was chosen near the beginning of the first period to capture muscle activities in fresh state (minimal fatigue). The second epoch was located at the middle of the second period of the force signal and expected to indicate moderate muscle fatigue. The third epoch was taken near termination of the contraction when the individuals were severely fatigued. Because participants sustained 30% MVC force throughout, it would be expected that the average force for each of the three periods would be similar (see force traces in Figure 1). Force variability was also evaluated during the three periods to assess steadiness of the force profile. It was quantified by calculating coefficient of variation (SD of the exerted force/mean force).

#### EMG analysis

All EMG signals were visually inspected to ensure they were artifact free. Similar to force recordings, EMG of the entire sustained contraction in each subject was evenly divided into initial, middle, and final periods (Figure 1). The three periods (minimal, moderate, and severe fatigue) of the force recording and EMG signals of the three tested muscle were defined with the same procedure. A 10-s epoch of EMG data corresponding to the 10-s epoch of force in each period was segmented in each muscle for further EMG analysis. The reason for analyzing EMG in shorter epochs instead of averaging them across each entire period was to fatigue period (minimal, moderate, or severe). Averaging data of entire period would obscure differences among the three fatigue stages.

EMG amplitude in each epoch was quantified offline by calculating the root mean square (RMS) for each muscle in each subject. The RMS is a standard method widely used for EMG amplitude quantification [19], [20]. We normalize each individual's EMG amplitude to his/her own MVC EMG amplitude. Because CRF patients' MVC force was further away from the true maximal force than healthy controls [9] and their MVC EMG values may also be far different from the true maximal value, which may make the normalized EMG more variable, we also analyzed absolute EMG RMS amplitude. Spectral content of the myoelectric signals was determined offline in each of 1024-ms windows (0.976 Hz frequency resolution) without overlap within each EMG epoch using Fast Fourier Transform (FFT) algorithm. Mean power frequency (MPF) was calculated in each 1024-ms window using SPIKE 2 analysis software (Cambridge Electronic Design, Cambridge, UK), and subsequently averaged for each of the three EMG epochs.

### Statistical Analysis

Data were analyzed using SPSS statistical package (SPSS 14.0, SPSS Inc., Chicago, IL, USA) and presented as mean and standard deviation (SD). To determine whether the parameters of force and EMG satisfied conditions for a normal distribution, the Shapiro-Wilk test was used. A three-way mixed ANOVA (3 Fatigue stages x 3 Muscles x 2 Subject groups, with repeated measures on the first two factors) was applied to compare differences among the fatigue stages and tested muscles between two groups (CRF and control) for EMG parameters. However, two-way mixed ANOVA was used to compare differences among the fatigue stages (3) between two groups for force of the sustained contraction and force variability. A contrast was used to find specific differences between fatigue stages and muscles. The Independent- Samples T Test was used to examine, whether the values of the endurance time, BFI scores and force of the MVC differed between CRF and control groups. Level of statistical significance was *P*≤0.05.

## Results

### Endurance Time, Force for Maximal Voluntary Contraction and Sustained Contraction

The endurance time was significantly shorter (*P*<0.01) for the CRF (332±134 s) compared with control (510±123 s) groups. CRF patients were weaker (*P*<0.05, MVC force was 191±71 N in CRF vs. 245±76 N in controls). Because the sustained force (30% MVC) was based on each subject's own MVC force, CRF patients sustained a smaller absolute force for a significantly shorter time. The elbow flexion MVC force was significantly lower in CRF than control subjects before and after the motor fatigue task (*P*<0.05) and in both groups the MVC force was significantly reduced (17.6% in CRF and 14.2% in controls) after the fatigue task (*P*<0.05). Furthermore, there was no significant difference between groups in the absolute and relative [%] reductions of the MVC force after the fatigue task (respectively *P* = 0.399, *P* = 0.859).

Target force for three periods of the sustained contraction did not change (*P*>0.1); force values for the first (minimal fatigue), second (moderate fatigue), and last (severe fatigue) periods were 30.2±2.6%, 29.7±2.2%, and 29.1±2.5% MVC force, respectively for the CRF patients and 30.7±3.1%, 30.4±2.9%, and 28.7±3.3%, respectively for controls. We found the effect of fatigue stage on the force variability was statistically significant (*F*(2, 44) = 17.42, *P*<0.001). However, the force variability was not different between groups at all three stages (*P* = 0.761). The coefficient of variation increased significantly (*P*<0.05) from periods 2 to 3 and from 1 to 3 in both groups (from 0.054±0.028 minimal fatigue, 0.052±0.036 moderate fatigue to 0.139±0.100 severe fatigue for the CRF patients, and from 0.042±0.018 minimal, 0.044±0.018 moderate to 0.104±0.067 severe for the controls).

### EMG Amplitude during Sustained Contraction

We found the effect of fatigue stage on the normalized EMG amplitude to be statistically significant (*F*(2.23, 0.09) = 24.72, *P*<0.001). The main effect of muscle on the normalized EMG amplitude was not significant (*F*(0.279, 0.106) = 2.629, *P* = 0.83). We did not find significant interaction between fatigue stages and muscles (*F*(0.012, 0.007) = 1.663, *P* = 0.166). The normalized EMG RMS value was not different between the groups at all three stages in the three tested muscles (*P* = 0.235). Furthermore, because normalized value of the RMS EMG (to RMS of subjects' own MVC) exhibited a similar pattern of changes between fatigue periods for both group with the absolute value of EMG amplitude, we decided to use the absolute value of EMG amplitude for further analysis. Furthermore, the MVC EMG RMS values were not different between the groups (CRF group: BB = 283±182, BR = 245±157, TB = 40±15 µV; Control group: BB = 294±182, BR = 342±152, TB = 55±16 µV).

**Figure 2:**
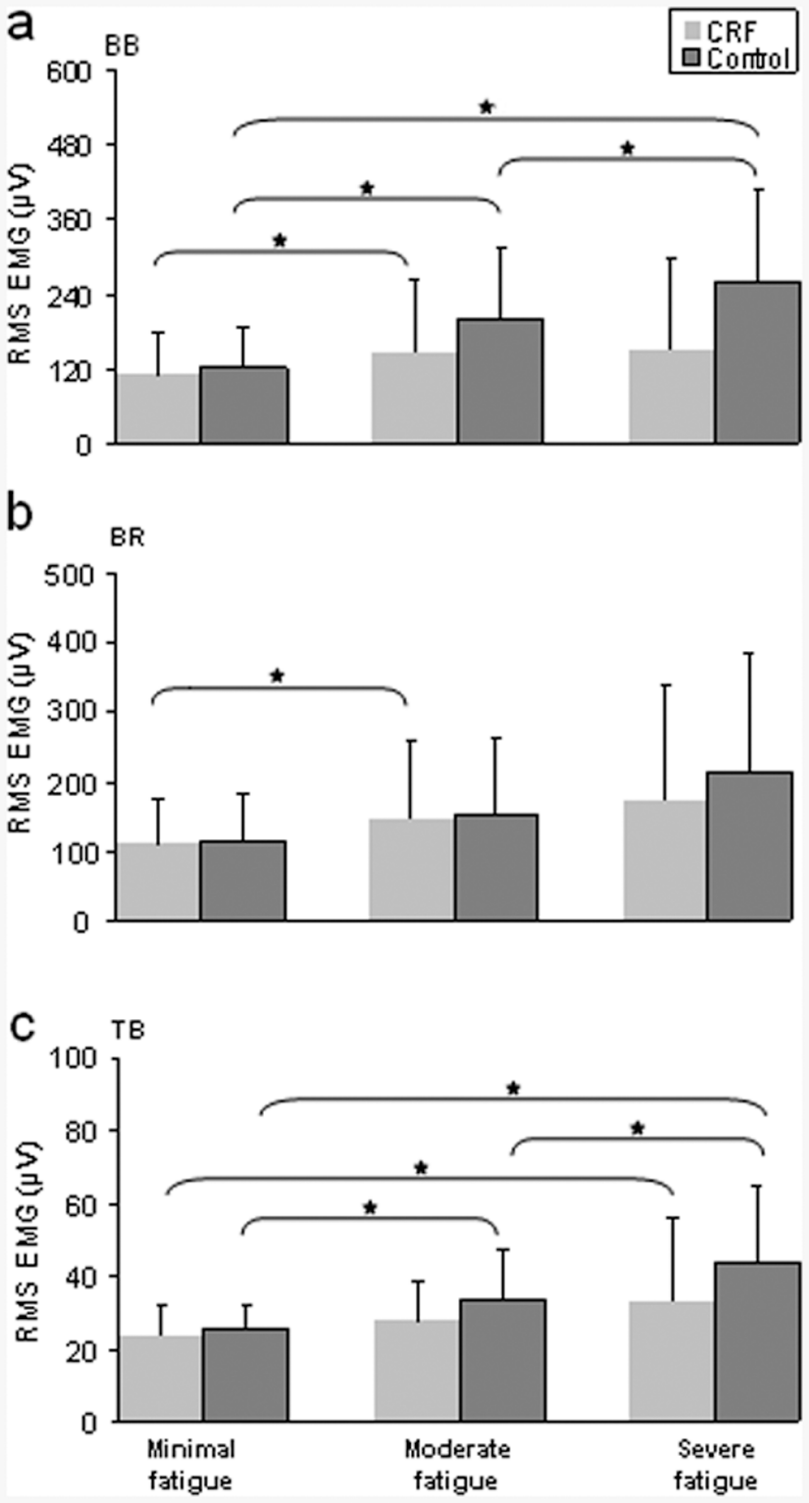
Group results of EMG amplitude (RMS in µV) in biceps brachii (BB), brachioradialis (BR), and triceps brachii (TB) muscles at stages of minimal, moderate and severe fatigue. CRF group showed smaller increases in EMG amplitude at moderate and severe fatigue stages especially for the BB (biceps brachii) and TB (triceps brachii) muscles, indicating a lower level of muscle fatigue. (A) The EMG amplitude in biceps brachii. (B) The EMG amplitude in brachioradialis. (C) The EMG amplitude in triceps brachii. * - Significant differences between fatigue stages, *P<0.05*.

Factorial mixed ANOVA for the absolute value of EMG amplitude during sustained contraction showed a significant main effect of fatigue stage (*F*(1.08, 23.73) = 11.32, *P*<0.001), muscle (*F*(2, 44) = 23.01, *P*<0.001) and the interaction between fatigue stages and muscles (*F*(1.45, 31.93) = 7.19, *P*<0.001). EMG amplitude was anticipated to increase to compensate for muscle fatigue as more motor units and muscle fibers are recruited to sustain the same force [21], [22]. The EMG RMS value increased significantly (*P*<0.05) from periods 1 to 2 to 3 in controls for the biceps brachii (BB) muscle. EMG amplitude during the same time periods changed modestly for the CRF group; a significant increase (*P*<0.05) was only seen from periods 1 to 2 (Figure 2, top panel). For the brachioradialis (BR) muscle EMG amplitude did not show substantial changes with the level of fatigue (only the CRF group exhibited a significant increase (*P*<0.05) from the periods 1 to 2; Figure 2, middle panel). The triceps brachii (TB, elbow extensor) muscle exhibited a similar pattern of change in the EMG amplitude with the BB muscle (Figure 2, bottom panel). The EMG RMS value was not different between the groups at all three stages in the three tested muscles (*P*>0.1).

### EMG Frequency during Sustained Contraction

**Figure 3:**
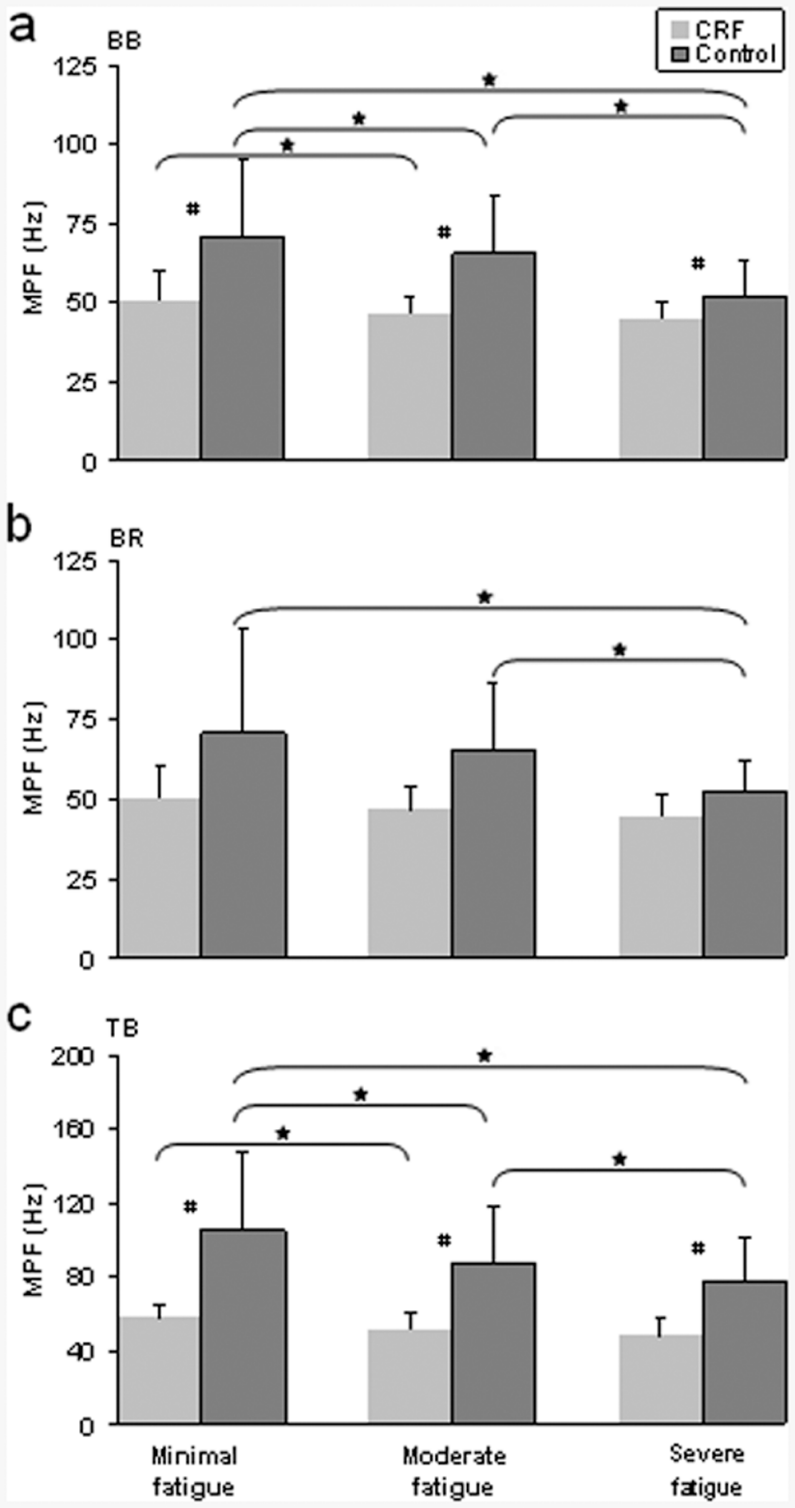
Group results of EMG mean power frequency (MPF) in biceps brachii (BB), brachioradialis (BR), and triceps brachii (TB) muscles at stages of minimal, moderate and severe fatigue. CRF group showed a lower degree of MPF reductions at moderate and severe fatigue stages especially for all 3 muscles, suggesting less muscle fatigue. MPF in CRF patients at minimal fatigue was similar to MPF at severe fatigue in controls. (A) The EMG mean power frequency in biceps brachii. (B) The EMG mean power frequency in brachioradialis. (C) The EMG mean power frequency in triceps brachii. * - Significant differences between fatigue stages, *P<0.05*; # - Significant differences between CRF patients and healthy controls *P<0.05*.

Statistically significant effects of fatigue stage (*F*(1.15, 25.26) = 13.08, *P*<0.001), muscle (*F*(1.24, 27.31) = 22.17, *P*<0.001) and interaction of both (*F*(2.94, 64.60) = 3.62, *P*<0.05) were found for the EMG MPF. It would be expected that EMG MPF would decrease with muscle fatigue as a result of fatigue-related physiological adaptations [17]. EMG MPF decreased significantly (*P*<0.05) from periods 1 to 2 to 3 in controls for the BB muscle and only changed moderately in this muscle for the CRF group; a significant reduction (*P*<0.05) in the MPF was only seen from periods 1 to 2 in CRF (Figure 3, top panel). For the BR muscle, the MPF did not show substantial changes with fatigue (*P*>0.05, Figure 3, middle panel). The triceps brachii (TB, elbow extensor) muscle exhibited a similar pattern of MPF change with the BB muscle (Figure 3, bottom panel). The EMG MPF was significantly lower (*P*<0.05) in the CRF group as compared to the controls for the BB and TB in three fatigue period (Figure 3).

### Peak Twitch Force (PTF) before and after Sustained Contraction

PTF declined significantly (*P*<0.05) in controls (26.4±8.0 N before and 18.5±7.7 N after sustained contraction, a 30% reduction) but the PTF change was not significant (*P* = 0.188) in CRF patients (20.4±5.5 N before and 17.0±4.4 N after sustained contraction). PTF is an index of force generation capability (FGC) of muscle and decreases with muscle fatigue [6], [11]. No significant differences were noted in the PTF between the CRF and control groups before (*P* = 0.080) and after sustained contraction (*P* = 0.606).

## Discussion

It has been shown that cancer survivors with fatigue symptoms undergo a greater level of central fatigue when engaging in typical prolonged motor activities [9]. In Yavuzsen et al. [9], a supramaximal-intensity electrical stimulus pulse applied to the biceps brachii muscle at the time CRF patients were about to fail a sustained elbow flexion contraction at 30% MVC demonstrated substantial evoked force. Despite their best effort to increase the muscle activation (recruiting additional motor units and increasing discharge rate of the active motor units) to sustain the contraction, additional force was elicited by the stimulation. This observation suggests that the patients experienced greater central fatigue than healthy controls [6]. The current study was designed to test the hypothesis that CRF patients would experience less muscular fatigue because of intensified central fatigue. The EMG data of myoelectrical manifestation of fatigue in CRF group being not as prominent as in controls support this hypothesis. EMG amplitude in CRF did not increase as much as in controls with fatigue. The PTF and EMG MPF in CRF did not decline as much as in controls with fatigue. Furthermore, individuals with CRF perceived exhaustion substantially sooner than the controls with similar changes of force variability by exerting the same relative (and smaller absolute) force. It is possible that significantly shorter endurance time for the CRF compared with control groups was related to more metabolic waste accumulation in the muscle of the control group. This could result in more prominent muscular fatigue in the control group. Furthermore, past studies [5] have reported neuromuscular abnormalities, including increased resting energy consumption and impaired muscle protein synthesis, adenosine triphosphate (ATP) generation, intracellular calcium flux, alterations in ryanodine receptors (RyRs) expression and higher levels of proinflammatory cytokines (interleukin-1 receptor antagonist, soluble TNF receptor type II, and neopterin) in CRF patients. The results seem to suggest that muscle tissue is directly involved in the pathogenesis of CRF and might contribute to impairment of neuromuscular efficiency during a prolonged motor task.

The standard deviation values (error bars) of the MPF measurements in the CRF patients were very similar among the three muscles and across the three time periods. This observation indicates low variability in MPF, regardless of the muscle examined or fatigue stage. Thus, if physiological conditions of the muscle are impaired by cancer or its treatment, the level of impairment seems to be similar among CRF patients. These data suggest that myoelectrical manifestation of fatigue in CRF is not as prominent as in controls. Furthermore, these data imply that subjective feeling of fatigue of patients with CRF in performing a prolonged voluntary muscle contraction does not correlate well with myoelectrical manifestation of muscle fatigue.

### EMG Amplitude

The EMG amplitude of the two elbow flexors and extensor near the time of exhaustion in CRF patients did not increase to the extent of that of healthy controls (Figure 2). As the muscle fatigues, the force-generating ability per motor unit/muscle decreases [11], [28]; as a result, more motor units need to be recruited (activated) as contractile failure occurs in those units already active to sustain the same load [17], [18], [29]. This would be reflected in increases in EMG amplitude. Furthermore, the interaction between fatigue stages and muscles was significant. The EMG amplitude was anticipated to increase to compensate for muscle fatigue as more motor units or muscle fibers of the synergist muscles were recruited to sustain the same force. Changes in surface EMG activity may reflect changes in motor unit recruitment strategy by the CNS (the increase of motor unit synchronization) and/or peripheral changes, such as impairments in neuromuscular transmission or action potential propagation along the muscle fibers. Our findings of smaller increases in EMG amplitude and substantial twitch interpolated force near exhaustion in CRF suggest reduced ability of the central nervous system to recruit motor units to maintain the contraction, which resulted in earlier task failure and a lower level of muscle fatigue indicated by insignificant changes in muscle contractile properties [8] and lower myoelectrical manifestation of fatigue in the CRF patients. Greater central fatigue has previously been observed in aging [19], [30], multiple sclerosis [31], and chronic fatigue syndrome [32].

One possible explanation for CRF patients failing the motor fatigue task much sooner than controls with smaller increases in EMG amplitude could be that they gave a lower general effort in performing the motor task especially near the time of task failure, perhaps in an unconscious effort to conserve energy. Although we could not completely rule out this possibility, we believe it was low as all the subjects received the same instructions and motivated to maintain the contraction by the same method. Most probably the patients' neuromuscular system was impaired by cancer and/or its treatment that might have interfered normal central regulation or homeostasis of the body systems, resulting perhaps abnormal sense of effort to lead to sooner task failure [33].

Potential mechanisms contributing to the smaller EMG augmentation in CRF near the time of task failure include reduction in descending drive to muscle from the motor cortex, increased inhibitory neuron activities [6], and/or reduced neuromuscular junction propagation (NMJP) function [34]. The study by Yavuzsen et al. [9] suggests both descending drive and NMJP reductions are possible in CRF. Central command originates from supraspinal centers and spinal motor neuron pool projects to muscle. Suboptimal output from the motor cortex during muscle fatigue has been reported [30], [34], which suggests fatigue-related weakening of descending input. Similarly, muscle fatigue dampens spinal motor neuron excitability [35], [36] and this together with impaired descending drive reduce the ability to increase muscle activation during fatiguing contraction. EMG amplitude can indirectly be influenced by negative feedback from small-diameter group III, IV afferents which inhibit cortical and spinal motor neurons [37]. However the role of group III and IV muscle afferents in controlling flexor motoneuron pool is not clear during fatigue. Martin et al. [38] suggest that the motorneuron pool of the elbow flexor muscles is not inhibited by fatigue-sensitive afferents during a fatigue muscle contraction (100% MVC) and changes in other reflex pathways may be relevant (in particular, reduction in muscle spindle firing rates and changes of the intrinsic motoneuronal properties). It is expected that influence of the negative feedback on muscle EMG in CRF would not be as severe as in controls (because of less muscle fatigue in CRF). All the aforementioned mechanisms that diminish EMG amplitude have not been excluded in CRF and remain a viable direction of research in searching for mechanisms that contribute to CRF.

### EMG Frequency

The mean power frequency (MPF) in CRF did not decline as much as in healthy controls (Figure 3). As muscle fatigue progresses, the MPF or median frequency of the EMG signals shifts to a lower frequency on EMG power spectrum [12]. EMG power of frequency spectrum changes are caused by many factors, including muscle activation level [39], action potential conduction velocity (APCV) that propagate along the muscle fiber [40], discharge rate of motor unit action potentials [41], motor unit synchronization [42], and temperature [14]. Among these, changes in the APCV perhaps have the strongest influence on the frequency power spectrum [43]. Muscle fatigue lowers the APCV [24], [44]. Numerous studies have reported shifting of the MPF or median frequency to lower frequencies under conditions of muscle fatigue [19], [20], [24]. Separating the contribution of the slowing of motor unit action potentials that is a variation of their conduction velocity from synchronization of motor units by the central nervous system to increase the mechanical output is limited using the mean power frequency [45], [46]. The significant interaction between fatigue stages and the MPF values of the muscles in both groups reveals differential adaptations in the MPF between the synergist and antagonist muscles as fatigue progressed. However the lack of leftward shifting of the MPF on EMG frequency power spectrum near the end of the sustained contraction in CRF indicates a lower level of myoelectric manifestations of fatigue in the patients compared with healthy controls. Both the EMG frequency and amplitude data that make a global estimation of myoelectric manifestations of fatigue suggest limited neural adjustments made during the fatigue task in CRF patients.

### Fatigue between Synergist and Antagonist Muscles

In all three muscles, the EMG amplitude increased significantly in CRF patients from the contraction periods 1 to 2 but not from periods 2 to 3 (Figure 2). This could be explained by greater central fatigue in CRF during period 3, which is considered as part of the neuromuscular system adjustment; after period 2, the rapid increase in central fatigue lead to difficulty in further activation of muscles. For MPF of the EMG, although the controls exhibited a significant decline from the periods 1 to 2, 2 to 3, and 1 to 3 in the BB and TB muscles, MPF in CRF also showed significant changes from periods 1 to 2 and 1 to 3 in the same two muscles. MPF may be a more sensitive measure to detect subtle physiological changes in the muscle with minimal to moderate muscle fatigue. Indeed, Bilodeau et al. [20] demonstrated more sensitive detection of muscle fatigue by MPF than amplitude using surface EMG at minimal-to-moderate fatigue levels.

It is worth noting that the MPF value in the time period 1 (minimal fatigue) in CRF was similar to the MPF in period 3 in controls; this was particularly true for the BB and BR muscles. This observation seems to indicate that the neuromuscular adjustment in the fresh state in CRF resembles fatigued conditions in controls. For all three muscles, the MPF in CRF was significantly lower than controls in all three time periods. Together, the data presented in Figure 3 strongly suggest a pathologic state in the neuromuscular system in CRF. The MPF is an index providing a global estimation of EMG fatigue, being sensitive to both fatigue-related variations of conduction velocity and motor units synchronization [45], [46]. The lower MPF in CRF could be contributed by slowed muscle fiber action potential conduction velocity and an increase in centrally-regulated motor unit synchronization even in a fresh, non-fatigued stage. It seems that CRF patients had worsened central fatigue and difficulty recruiting more motor units with high discharge rates and/or new motor units to replace failing ones to participate in the sustained contraction. The absence of these motor units in the motor task would also lower the MPF and amplitude. Figure 3 also shows that the standard deviation values (error bars) of the MPF measurements in the CRF patients were very similar among the three muscles and across the three time periods. This observation suggests low variability of MPF, regardless of the muscle examined or fatigue stage. Thus, if physiological conditions of the muscle are impaired by cancer or its treatment, the level of impairment seems to be similar among CRF patients.

The TB muscle is an antagonist of elbow flexion and exhibited a similar pattern of the EMG (amplitude and MPF) alteration in both groups during the sustained contraction compared to the BB muscle, the major elbow flexor. Normally, the antagonist activation level is relatively low (see Figures 1 and 2) but it changes with the agonist muscle as it stabilizes the joint and shows sign of “fatigue” [29], [41]. The increase in TB EMG amplitude apparently was a result of recruitment of motor units and/or increasing discharge rate of the activated motor units. The decline in the MPF in this muscle could primarily be attributed to slowing of conduction velocity of action potentials of motor units that were active since the beginning of the sustained task.

For the control subjects, fatigue measured by EMG amplitude and MPF in the BB muscle was more prominent than that in the BR muscle (Figures 2 and 3). During the contraction subjects sat with the forearm in a neutral position and elbow joint at 100°. This elbow joint angle is considered as optimal biomechanical position for the BB (for BR in ∼70°). According to the study of Van Zulen et al. [47], muscle with the optimal biomechanical condition receives higher level of neural drive. This explains why the BB is a more active muscle than the BR for elbow flexion. For this reason, during sustained elbow flexions the BB could be more substantially used than the BR muscle.

### Force Steadiness

The standard deviation of the force in a given time window divided by its mean value is named coefficient of variation and is used to assess steadiness or amplitude of force fluctuation. Force steadiness is reduced in fatigue, and experimental [48] and simulation [49] studies indicate that the increase in force fluctuation (quantified by coefficient of variation) is related to an increase in the fluctuation of the common drive to the muscle, which at least in part originates from supraspinal centers. In our study the coefficient of variance increased significantly from moderate to severe fatigue stages in both groups and the force variability was not different between groups at all three stages. The fact that the level of force fluctuation appears to be the same for CRF and control groups with less prominent myoelectrical manifestation of fatigue for CRF may imply a higher level of central fatigue for this group. Semmler [50] and Yao et al. [51] noted that increased motor unit synchronization contributes to larger force fluctuation. Moritz et al. [52] suggest that the discharged rate variability was a factor affecting force fluctuation. Although synchronization of motor-unit activity is commonly suggested as a factor that influences quality of force output during fatiguing contractions, direct evidence of changes in synchronization during fatiguing tasks is controversial. However, some indirect measures provide evidence of increased motor-unit synchronization during fatigue of the biceps brachii [53] and an increased common neural drive input across synergistic muscles [54]. It has been shown that motor unit recruitment and synchronization is related to higher force fluctuations in simulated muscle contractions [51], [55]. The results of coefficient of variation of the sustained force in the current study provide experimental evidence supporting a decreased level of quality of the force profile (reduced force steadiness in fatigue) that was indirectly related to an increase in the fluctuation of the common drive to the muscle, alternation of the motor units activation strategy (recruitment and firing rate of MUs) associated with central fatigue.

### Peak Twitch Force

PTF decreased significantly in controls but not in CRF patients. This finding suggests that muscle fatigue in CRF was less severe as in controls. Thus, a less prominent myoelectrical manifestation of fatigue in CRF (measured by EMG amplitude and frequency parameters) was also confirmed by lack of impairment within the muscle through PTF analysis. Reduced force generating capability is direct evidence of muscle fatigue and it is typically indicated by a decrease in electrical stimulation-evoked PTF in humans [11]. Because the evoked PTF was not influenced by voluntary activation, the mechanism contributing to PTF reduction is likely attributed to impairment in excitation-contraction (E-C) coupling [56] that includes functional events between generation of an action potential along muscle fiber membrane and force produced by the fiber [57].

### Weaknesses

It is well know that myoelectric manifestation of fatigue during voluntary contractions appears to be influenced by a number of factors including peripheral and central components of the neuromuscular system. The adjustments that occur during a sustained submaximal fatigue contraction result in changing distribution of action potential sizes and the amount of amplitude cancellation, which could alter the relation between the neural activation of muscle and surface EMG [58], [59]. Both descending command and NMJP function are impaired in CRF [9] and could have contributed to the EMG increase and perhaps MPF decrease. This study was unable to determine relative contribution of each of the two to the EMG signal changes. However, given that any changes occurring at and above neuromuscular junction are considered central and both mechanisms contribute to central fatigue.

The ages between CRF (55 yrs) and controls (47 yrs) seem different but did not reach statistical significance because of relatively large age variations. Such a small discrepancy in age between groups does not seem to contribute significantly to EMG amplitude and MPF differences. Bilodeau et al. [19] reported such differences typically had an age discrepancy at least several decades between young and old groups. Although, the neuromuscular adjustments in aging begin at the age of 60 and may therefore affect the CRF-group more than the control (because CRF subjects were older), we would expect more myoelectric manifestation of fatigue in CRF group under this condition. Muscle mass is expected to be smaller in CRF compared to controls as the patients were significantly weaker. Muscle size can affect EMG amplitude but it might have little effect on EMG results of this study. The sustained force was relative to the MVC force for patients and controls and therefore, both groups should have similar abilities to manage muscle EMG (both groups could monitor EMG from ∼30% MVC to 100% MVC). Moreover, EMG signal comparisons were made across the three time periods within each group and between-group muscle mass difference is not relevant to within-group comparisons.

Medication (opioid) taken by CRF patients may affect muscle activation/motor unit recruitment and EMG amplitude and MPF. The drug blocks “central governor” and afferent signals to the central nervous system and limits peripheral muscle fatigue development in humans [60], and it can lead to greater muscle recruitment in athletes. However, if the drug effect on EMG signals is facilitation, then the CRF patients should have had greater EMG amplitude increases than controls who did not have the medication. In palliative situations, corticosteroids can temporarily improve patients' physical activity; however, prolonged use of the drugs may induce myopathy, which in turn might worsen CRF [61]. Treatment with different medications may have effects on patients' physical performance [61], [62] and more research is needed to better recognize effects of patients' medication on neural and muscular system function.

### Conclusions

In this study, the EMG amplitude and MPF, two standard physiological assessments for evaluating muscular fatigue plus the twitch force data, suggest that patients with cancer related fatigue do not undergo as much muscle fatigue as healthy individuals even they feel their muscles are “exhausted” by the physical exertion. The early arrival of perceived physical exhaustion and lack of prominent electromyographic changes during a prolonged, submaximal motor task in cancer survivors with fatigue symptoms suggest indirectly that central nervous system fatigue plays a more important role in limiting endurance-type motor performance in these patients.
